# Primary *Candida guilliermondii* Infection of the Knee in a Patient without Predisposing Factors

**DOI:** 10.1155/2012/375682

**Published:** 2012-02-28

**Authors:** Gun Woo Lee, Tae-Hun Kim, Jung-Hwan Son

**Affiliations:** Department of Orthopedic Surgery, Gospel Hospital, Kosin University, 34 Amnam-Dong, Seo-gu, Busan 602-702, Republic of Korea

## Abstract

Isolated primary candidal infection of joint is extremely rare, with only a few reported cases. It occurs as a result of accidental implantations of fungus during traumatic procedures, such as surgery, and is usually reported in patients with predisposing factors such as immunosuppression, malignancy, and drug abuse. If left untreated, irreversible deformity and pain with severe osteoarticular destruction occur. Thus, early diagnosis and treatment are important. This paper presents a case of 72-year-old man with primary *C. guilliermondii* infection of knee joint without predisposing factors and previous traumatic procedures, who was misdiagnosed with advanced degenerative osteoarthritis. Our case is the second case of primary *C. guilliermondii* arthritis of knee to be reported in the English-language literature and the first to be successfully treated with total knee arthroplasty following IV amphotericin B and oral fluconazole. Primary candidal infection of joint is generally asymptomatic or involves only mild pain and swelling in the affected knee. Thus, although the majority of knee joint infections are of a pyogenic or tuberculous origin, if a patient complains of mild pain and swelling in the knee and has mild signs of infection, the possibility of fungal infection should be considered.

## 1. Introduction

Candidal infection of knee joint is rare. It is primarily reported in immunosuppressed patients as a sequela of prior episodes of transient candidemia or as a result of disseminated disease [1–3]. In the majority of cases, candidal arthritis is the result of hematogenous dissemination. Primary candidal infection of joint is extremely rare, with only a few reported cases. It most often occurs as a result of accidental implantation of fungus during traumatic procedures, such as surgery, intra-articular injections, or implantation of infected joint prostheses [4–6], and also in human-immunodeficiency-virus-(HIV)-infected intravenous drug users [7, 8] and heroin addicts [9]. While some *Candida* species, including *C. albicans, C. krusei, C. parapsilosis, C. tropicalis, C. glabrata, *and* C. lusitaniae*, have been incriminated in the etiology of osteoarticular infections [10–14], *C. guilliermondii* joint infection has been reported only once, by Graham and Frost [15]. However, the case reported by Graham and Frost occurred in the setting of rheumatoid arthritis, which is one of the minor predisposing factors for candidal infection of joint.

Here we report a case of primary *C. guilliermondii* infection of the knee joint in a patient without predisposing factors that was successfully treated by total knee arthroplasty following intravenous amphotericin B and oral fluconazole. To the best of our knowledge, our case is the second case of primary *C. guilliermondii *infection of joint reported in the English-language literature, and the first in a patient without predisposing factors. Additionally, ours is the first reported case to be successfully treated by total knee arthroplasty following intravenous amphotericin B and oral fluconazole in case of candidal joint infection.

## 2. Case Report

**Figure 1:**
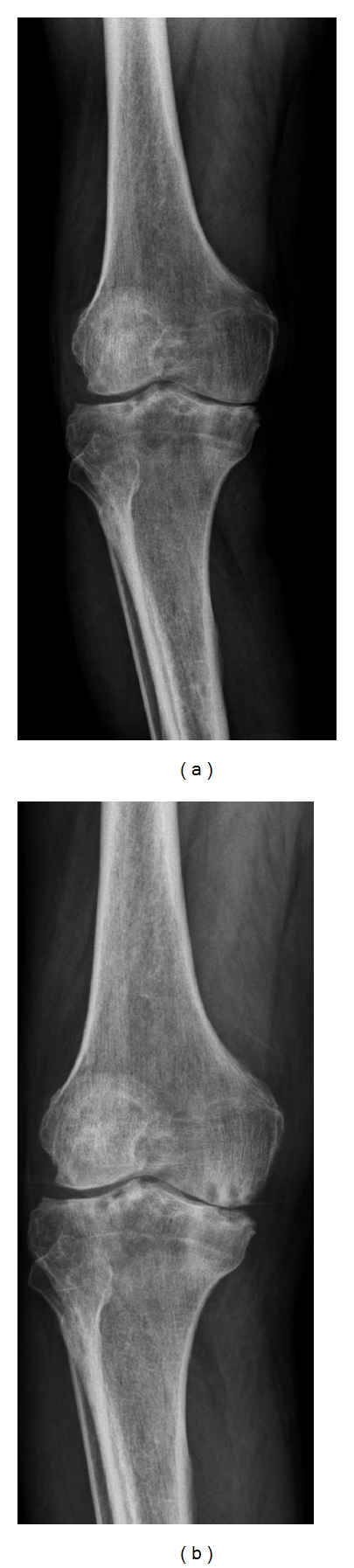
(a) Standing knee radiograph showed multiple patched subchondral lesions of the distal femur and proximal tibia, joint space narrowing, and definite osteophyte. (b) After disappearing of the infection sign, standing knee radiograph showed aggravation of intra-articular deformity.

A 72-year-old man presented to our clinic with a 1-year history of pain and swelling of the right knee. He had been having intermittent pain in both knees for 15 years. Medication for mild degenerative arthritis had been recommended, but the patient refused treatment. A year before he presented to our clinic, he visited another clinic due to the aggravation of pain and swelling of the right knee and was diagnosed with severe degenerative arthritis by radiologic and physical exam, and a decision was made to perform total knee arthroplasty. During the operation, severe inflammation and an abscess were found inside the knee joint; therefore, bacterial culture was obtained and arthroplasty was not performed. After the operation, he was diagnosed by culture with unknown-originated joint infection and transferred to our clinic. The medical history was negative for rheumatoid disease, cancer, renal disease, and HIV. He was a nonsmoker and nondrinker and did not take any steroids or illegal drugs. On physical examination, the patient had signs of infection, including swelling and localized warmth, of right knee. Knee radiographs revealed multiple patched subchondral lesions of the distal femur and proximal tibia, severe joint space narrowing, and definite osteophytes (Figure 1(a)). These findings were suggestive of a degenerative knee joint (K-L grade IV).

**Figure 2:**
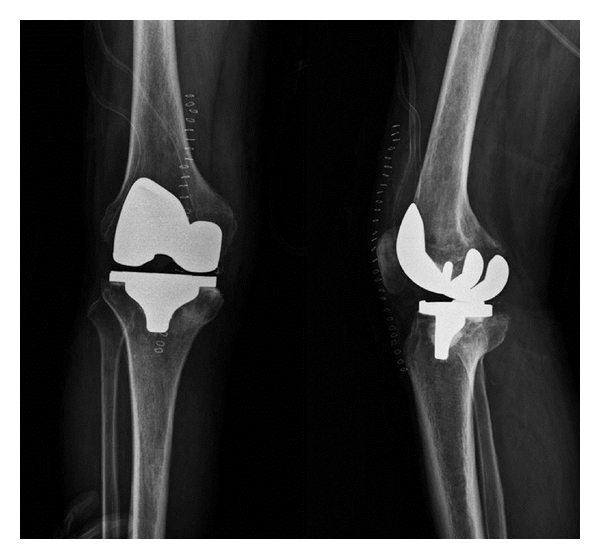
Primary total knee replacement with the Duracon prosthesis (Howmedica, Rutherford, NJ, USA) was performed.

**Figure 3:**
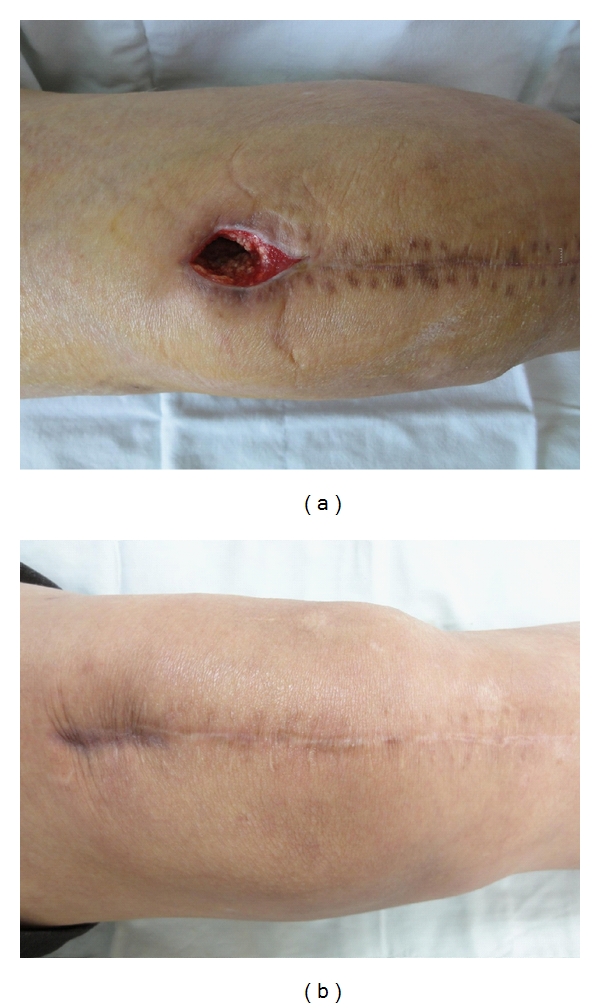
(a) Superficial infection at proximal operative site was noted at postoperative week 2. Debridement and closure were performed. (b) After 6 months of follow-up, there was no abnormal finding such as inflammation, and he has led a normal life.

The laboratory results were as follows: white blood cell (WBC), 11,200/mm^3^ (4–10 × 10^3^/mm^3^); erythrocyte sedimentation rate (ESR), 97 mm/hr (0–15 mm/hr); C-reactive protein (CRP), 19.2 mg/dL (0–5 mg/dL). Tests for anti-nuclear antibody (ANA), rheumatoid factor (RF), and HLA B_27_ were all negative. The joint fluid cytology findings were as follows: appearance, cloudy and reddish; WBC, 17,200/mm^3^ (0–200/mm^3^) with 85% neutrophils and 10% lymphocytes. The Gram stain and acid-fast bacteria (AFB) stain showed no bacteria or tuberculosis. *Candida guilliermondii* was cultured in the joint fluid on hospital day 7 and was confirmed on hospital day 8. Amphotericin B (0.7 mg/kg/day) was given intravenously. Two weeks later, CRP had normalized. After 4 weeks, the amphotericin B was discontinued and oral fluconazole (400 mg/day) was prescribed for 6 months. The last culture of joint fluid was negative for *C. guilliermondii,* and the patient had no complaints of pain or swelling. The ESR/CRP was continuously maintained at normal state. After the inflammation had resolved, the patient had pain on the medial side of the knee joint with ambulation, and rechecking knee radiographs revealed aggravation of the patient's intra-articular deformity due to the candidal infection (Figure 1(b)). After 4 months, primary total knee replacement with a Duracon prosthesis (Howmedica, Rutherford, NJ, USA) was performed (Figure 2). The wound was daily dressed, and intravenous amphotericin was injected; however, redness and swelling were observed at the proximal operative site 1 week postoperatively (Figure 3).

 The patient was diagnosed with a superficial infection, and wound debridement and closure were performed. So far, after discharge, there were no abnormal findings or signs of infection.

## 3. Discussion

In our patient, infection of the knee joint occurred without predisposing factors, such as autoimmune disease, HIV, chronic steroid use, cancer, or drug abuse; therefore, most clinicians suspected pyogenic or tuberculosis infection and did not consider fungal infection because it rarely occurs in normal population [16]. Only a few cases of primary candidal infection have been reported. In our knowledge, primary candidal infection has been reported in less than 15 cases, mostly in neonates [17], septicemic patients, HIV patients, and drug abusers [7–9]. While only some *Candida *species have been incriminated in the etiology of osteoarticular infections, *C. guilliermondii* has been reported only one time, by Graham and Frost [15]. Graham reported that a patient with longstanding rheumatoid arthritis and myasthenia gravis presented with mild knee pain and swelling and suggested that the unusualness organism, of the apparent chronicity of the infection, and the ultimate success of the surgical treatment are features of this organism. However, that case occurred in a patient of rheumatoid arthritis with systemic corticosteroid, which is one of predisposing factors.

Disseminated *Candida* infection manifest with systemic symptoms including cutaneous and ocular lesions, so early diagnosis and treatment are possible. However, primary candidal infection of joint usually has mild, chronic course with little pain and few articular symptoms, leading to delays in diagnosis that can be as long as several years [14, 18]. Due to these ambiguous symptoms, primary candidal infection is often misdiagnosed as simple degenerative osteoarthritis of the knee, as in this case. However, if left untreated, irreversible deformity and pain with severe osteoarticular destruction occur. Thus, early diagnosis and treatment are important. Thus, if there are minimal infectious signs, careful physical examination and attentive analysis of blood and joint fluid should be conducted to avoid incorrect diagnosis and treatment. In addition, because the majority of knee joint infections are of a pyogenic or tuberculous origin, most physicians do not perform the study about fungus infection. Thus, if there is an ambiguous case, which shows mild pain, swelling, and infection sign, a study about fungal infection should be also done.

Definitive information on the treatment of native joint *Candida* infection is limited [19]. Both amphotericin B and fluconazole have been successfully used to treat infection with susceptible strains, as long as adequate drainage is provided [17]. Because successful treatment with amphotericin B is more commonly reported, we initiated treatment with intravenous amphotericin B, which was changed to oral fluconazole 4 weeks later. A long-term survey of *Candida *demonstrated that fluconazole has fewer side effects and better antifungal effects than amphotericin B [20]. Therefore, we prescribed fluconazole for 6 months. Six months later, neither signs of inflammation nor side effects were observed.

As determined by a search of Medline for articles between 1966 and January 2011, our case is the second case of primary *C. guilliermondii *infection of knee joint to be reported in the English-language literature and the first to be successfully treated with total knee arthroplasty following IV amphotericin B and oral fluconazole. Primary candidal infection of joint is generally asymptomatic or involves only mild pain and swelling in the affected knee. Thus, if a patient complains of mild pain and swelling in the knee and has mild signs of infection, the possibility of fungal infection should be considered.

## 4. Conclusion

Primary candidal infection of joint is extremely rare, with only a few reported cases. This infection is generally asymptomatic or involves only mild pain and swelling in the affected knee. However, if left untreated, irreversible deformity and pain with severe osteoarticular destruction occur. Thus, early diagnosis and treatment are important. Although the majority of knee joint infections are of pyogenic or tuberculous origin, if a patient complains of mild pain and swelling in the knee and has mild signs of infection, the possibility of fungal infection should be considered.
